# The psychiatric phenotype of 15q11.2-q13.3 duplications

**DOI:** 10.1192/j.eurpsy.2021.1908

**Published:** 2021-08-13

**Authors:** M. Budisteanu, S. Papuc, A. Erbescu, E. Andrei, I. Streata, M. Cucu, C. Iliescu, C. Anghelescu, D. Ioana, M. Ioana, F. Rad, A. Arghir

**Affiliations:** 1 Medical Genetics, University Titu Maiorescu, Bucharest, Romania; 2 Genetics Laboratory, ‘Victor Babes’ National Institute of Pathology, Bucharest, Romania; 3 Department Of Child Psychiatry, ‘Prof. Dr. Alexandru Obregia’ Clinical Hospital of Psychiatry, Bucharest, Romania; 4 Medical Genetics, 4. University of Medicine and Pharmacy, Craiova, Romania; 5 Psychaitry Research Laboratory, Obregia Clinical Hospital of Psychiatry, Bucharest, Romania; 6 Child And Adolescent Psychiatry, “Alexandru Obregia” Clinical Psychiatry Hospital, Bucharest, Romania

**Keywords:** intellectual disability, autism, 15q11.2-q13.3 duplications, phenotype

## Abstract

**Introduction:**

15q11.2-q13.3 region is prone to genomic rearrangements leading to both deletions and duplications. A wide spectrum of neuropsychiatric conditions, such as developmental delay/intellectual disability (DD/ID), autism, attention-deficit hyperactivity disorder, schizophrenia, epilepsy was reported in association with genomic imbalances of this region.

**Objectives:**

In this paper we report on 9 children carrying 15q11.2-q13.3 duplications.

**Methods:**

Seven boys and two girls, aged 15 months to 15 years, were included in the study. Genomic investigations were carried out by array-based comparative genomic hybridization (Agilent Technologies). In all patients the psychomotor development, dysmorphic features, neuroimaging and EEG anomalies were assessed. Psychologic and psychiatric evaluation was performed with specific tests.

**Results:**

The size of the duplications ranged from 9.65 Mb to 0.38 Mb. All patients presented speech delay. Autistic behavior and muscular hypotonia were detected in 8 out of 9 patients, DD/ID in 6. Two children presented epileptic seizures, in addition 4 other children had EEG anomalies. Facial dysmorphic features were observed in 5 patients. Neuroimaging studies showed anomalies in 4 children. The smallest region of overlap in our patient group harbors CHRNA7 gene, a candidate for the behavioral abnormalities.

**Conclusions:**

15q duplications encompassing CHRNA7 gene were associated with different neuropsychiatric features in our patients. Our results further support the association of 15q duplications with neuropsychiatric phenotypes, with clinical heterogeneity and variable severity, which is yet to be explained. Acknowledgment: The research leading to these results has received funding from the EEA RO NO Grant 2014-2021, the project contract No 6/2019.

**Disclosure:**

No significant relationships.

